# Can diary writing facilitate recovery: an auto-ethnography analysis

**DOI:** 10.1186/2050-2974-3-S1-O29

**Published:** 2015-11-23

**Authors:** June Alexander, Margaret McAllister, Donna Lee Brien

**Affiliations:** 1 Central Queensland University, Australia

## 

Diary writing may seem a simple self-help tool, requiring only pen, paper and time. However, the unwary diarist may become entrapped in self-defeating thoughts and anxieties, and be swept out in a rip-tide of self-sabotage and self-doubt. Regimented lists of rules that focus on calories, exercise and weight may ease anxiety momentarily, but also disconnect body from self. The distancing effect that writing may have for a person who is struggling with inner anxieties is also double edged – it can exacerbate dissociation from one's own body and personal control, or it can illuminate new understandings.

This presentation will draw upon an auto-ethnographic analysis to reveal that diary writing is a complex art. For the first author, the diary was both a constructive and destructive tool in the early years of a 40-year struggle with Anorexia Nervosa. Insights on this, and how diary-writing techniques assisted in reconnection with an authentic self, are discussed, showing that the diary offers an opportunity to be both life participant and observer. Findings indicate that, despite its dangers, guided diary writing can act as a lifebuoy between person and therapist.

